# Hydroxytyrosol and Potential Uses in Cardiovascular Diseases, Cancer, and AIDS

**DOI:** 10.3389/fnut.2014.00018

**Published:** 2014-10-27

**Authors:** Cristina Vilaplana-Pérez, David Auñón, Libia A. García-Flores, Angel Gil-Izquierdo

**Affiliations:** ^1^Department of Food Science and Technology, Centro de Edafología y Biología Aplicada del Segura, CSIC, Murcia, Spain; ^2^Department of Research and Development, Seprox BIOTECH, S.L., Madrid, Spain

**Keywords:** hydroxytyrosol, olive oil, phenolic compounds, disease, anti-oxidants

## Abstract

Hydroxytyrosol is one of the main phenolic components of olive oil. It is present in the fruit and leaf of the olive (*Olea europaea L.)*. During the past decades, it has been well documented that this phenolic compound has health benefits and a protective action has been found in preclinical studies against several diseases. Here, we review its bioavailability in human beings and several assays showing significant results related with cardiovascular diseases, cancer, and acquired immunodeficiency syndrome (AIDS). Mechanisms of action include potent anti-oxidant and anti-inflammatory effects, among others. The importance of hydroxytyrosol in protection of low-density lipoproteins and consequently its implication in the reduction of cardiovascular disease risk has been highlighted by the European Food Safety Authority, concluding that 5 mg of hydroxytyrosol and its derivatives should be consumed daily to reach this effect at physiological level. We discuss the potential uses of this compound in supplements, nutraceutic foods, or topical formulations in the disease risk reduction. Finally, we conclude that more studies are needed to sustain or reject many other health claims not yet fully documented and to validate these newly available hydroxytyrosol-based products, because it seems to be a good candidate to reduce the risk of diseases mentioned.

## Introduction

### Hydroxytyrosol and Mediterranean diet

Hydroxytyrosol (HOTYR) is a simple phenolic compound naturally occurring in olive and olive oil, the main source of fat in Mediterranean diet. Nowadays, it is very popular the benefits of this diet for the maintenance of human health and wellbeing. Anyway, the concept of Mediterranean diet may be misunderstanding, being necessary its clarification. It was originally defined as the dietary pattern found in some of the olive-growing regions in the Mediterranean Basin of the 1960s. Thus, a current simple accepted definition is closely related to the diet characteristics as source of separate types of fats. In this way, Mediterranean diet highlights by its high proportion in plant foods where the main source of fat is olive oil, resulting in a higher proportion of mono unsaturated/polyunsaturated fats relative to saturated ones (1).

The healthy benefits of the Mediterranean diet are popularly known and are supported by numerous studies, as the well-known “The Seven Countries Study,” which highlighted the cardiovascular disease related mortality in Mediterranean region compared with other countries with distinct dietary habits in the 1960s (2). This study can be considered as pioneer and opened a high number of researches on those foods with critical influence in the benefits of the Mediterranean diet. Among them, the PREDIMED trial (“Prevención con dieta Mediterránea”) is a large (7447 subjects), parallel-group, multicenter, randomized trial for a follow-up period of 4.8 years, whose participants were assigned to three dietary intervention: Med-diet supplemented with virgin olive oil, Med-diet supplemented with nuts, and low-fat diet. The results showed an important reduction of three of every thousand persons per year in the risk of major cardiovascular events among high-risk persons.

Among the separate foods taking part of the Mediterranean diet, olive oil is considered a crucial element, being one of the most traded and consumed product in the world directly related to the healthy attribution of this popular diet (1). In November 2004, the Food and Drug Administration (FDA) allowed the health benefits of olive oil due to monounsaturated fat in reducing the risk of coronary heart disease and recommended a daily intake of two tablespoon (23 g) (3).

The responsible parameters of the healthy attributions of olive oil and table olives remain poorly understood. Olive oil is mainly composed of two groups of compounds: saponifiable lipids (98.5–99% of the total composition) and an unsaponifiable fraction. The main triglyceride fatty acid is the oleic acid with 55–83% of the total fatty acid content. It also includes moderate amounts of palmitic and linoleic acids and a low percentage of stearic and linolenic acids. On the other hand, the unsaponifiable fraction contains a large variety of compounds responsible for stability and organoleptic characteristics of olive oil. This fraction consists of primarily of hydrocarbons (squalene, carotene, lutein), terpene compounds, sterols, phenolic compounds, and aliphatic alcohols among others. Phenolic compounds from olive oil include four different groups: simple phenols, i.e., tyrosol (TYR), hydroxytyrosol, polyphenols (flavonoids), secoiridoids (oleuropein), and lignans (4).

Although the major components of olive oil are its monounsaturated fatty acids, mainly oleic acid, we cannot attribute the beneficial effects of the Mediterranean diet only to these substances because other oils, such as rape-seed or canola oil are also rich in monounsaturated fatty acids and may produce the same benefit. For this reason, attention has focused on the phenolic compounds, which have showed a strong anti-oxidant capacity, of virgin olive oil that distinguish themselves from other types of fat. The amount of phenolic compounds present in olive oil greatly varies depending on cultivar, degree of maturation, climate, and manufacturing process (5). Thus, extra virgin olive oil, obtained from the cold pressing or centrifugation of the olives, is the one with higher content of total phenolic compounds. An early study showed a total phenolic content of 232 mg/kg in virgin olive oil versus 62 mg/kg values of refined olive oil (up to 80% less usually lost during the refining process) (6).

In this sense, olives, olive oil source, have higher amounts of phenolic compounds that extra virgin olive oil (oil with higher anti-oxidant capacity). Depending on the variety of olive and the type of processing, the phenolic content varies significantly. As an example, total phenolic content estimated in black olives was 16.40 g/kg dry weight, representing hydroxytyrosol 5.78 g/kg. Tyrosol, phloretic acid, dihydrocaffeic acid, acteoside, luteolin, and apigenin were also found. The green olives content was much lower with 4.48 g/kg of hydroxytyrosol and only traces of other phenolics (6, 7).

**Figure 1:**
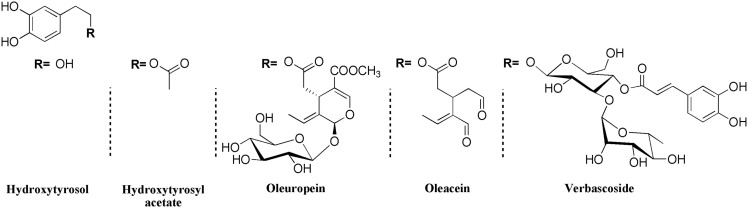
**Hydroxytyrosol and derivatives in olive and olive oil**. Adapted from Boskou (10).

**Figure 2:**
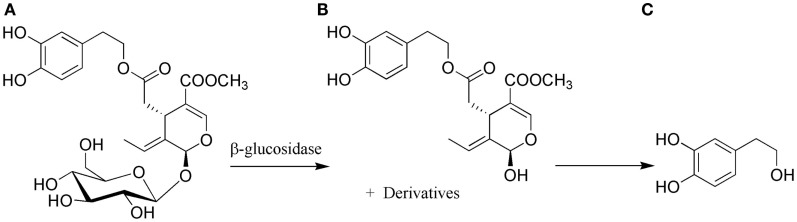
**(A)** Oleuropein-glycoside ripening olives and olive oil. **(B)** Oleuropein-aglycone. **(C)** Hydroxytryosol. Adapted from Charoenprasert et al. (12).

Hydroxytyrosol, 2-(3,4-dihydroxyphenyl)-ethanol (HOTYR), is a phenolic compound present in the fruit and leaf of the olive (*Olea europaea L*.), which belongs to the family Oleaceae, comprising species distributed throughout the temperate regions of the world, and essentially localized in the Mediterranean basin. Hydroxytyrosol is one of the main components of virgin olive oil, olive mill waste water (OMWW), and olive leaf extract (OLE), which was early identified as the strongest *in vitro* anti-oxidant potential among all the olive oil polyphenols (8). As mentioned above, the content of this compound is largely dependent on the oil quality. Amounts range from 14 mg/kg in extra virgin olive oil, up to <2 mg/kg (7). Other estimations increased the estimation to total phenolic content of olive oil in ranges from 487.5 to 1950 mg/l, and HOTYR specifically from 20 to 84 mg/l (9). Hydroxytyrosol in oil is found free, in acetate form or as a part of more complex compounds like oleacein, oleuropein, and verbascoside (10) (Figure 1). Oleuropein is responsible for the bitter taste of olives and decreases as the fruit ripens turning into unglycosylated form, oleuropein aglycone by enzymatic hydrolysis, and finally converted into hydroxytyrosol, being this one an indicator of maturation of the olives (11, 12) (Figure 2).

Hydroxytyrosol, with a molecular weight of 154.16 is a white powder with melting point around 55°C and is fairly soluble in water and polar organic solvents such as low-molecular weight alcohols. Like other polyphenols, it is easily oxidized in aqueous solutions unless adequate precautions, acid pH, and absence of oxidants, are taken. As it was noted at the beginning, HOTYR is considered by far the main simple phenolic compound of table olives (13) although the concentration depends on olive variety (11), maturation, and the processing method (7). Besides, the concentration of phenolic compounds is different when comparing table olives and raw fruit due to manufacturing process. For example, in the preparation process of the Californian-type black olives, levels of HOTYR and caffeic acid decreases in the darkening process (14). Therefore, the beneficial properties for human health found in olive oil could also be supposed and extended in table olives. Moreover, HOTYR was identified in olive pits as shown by a 2001 patent explaining the synthesis of this compound by a process of quick autohydrolysis (15). Another natural source of HOTYR is red wine (16), and although the concentrations found are lower than those found in olive oil, unsettling bioavailability studies reveal the emergence of higher quantities of HOTYR concentrations than those administered, in urinary recoveries after administration of red wine. An interaction between ethanol and dopamine leading to the formation of hydroxytyrosol was suggested as a possible explanation to this fact (17). Therefore, all these data reveal the relevance of HOTYR consumption by olive products, which are regularly consumed in the Mediterranean diet and ensure a daily contribution to the human health.

## Absorption and Bioavailability

The term bioavailability is commonly used to indicate the proportion of a substance that reaches the systemic circulation after oral administration considering both its absorption and its local metabolic transformation.

There are several assays dealing with the absorption of olive oil phenolic compounds both in animals (18, 19) and human beings. Visioli et al. (9) found a high correlation for TYR and HOTYR in human urine respect the ingested of different phenolic amounts in olive oil. The urine hydrolyzed using glucuronidase enzyme revealed the high grade of conjugation of HOTYR and an increasing conjugation degree in higher phenolic content oils was found. Miró-Casas (20) also demonstrated the absorption of hydroxytyrosol in a study with human beings after ingestion of olive oil. The recovery reached for hydroxytyrosol was 121.1% when was administered as a maintenance dose of 25 ml/day for 1 week. These data have been also found in other studies (17, 21, 22) and different explanations have been offered, like the hydrolysis of precursors also present in olive oil, long-term accumulation and dopamine metabolism. Studies using different techniques to determine the amount of absorbed hydroxytyrosol as isotopic labeled (19) estimated absorption around 99% of totally intake in oil and 75% in aqueous solution, being <3% in fecal content. Around 55–66% of absorption was found too with aqueous supplements in ileostomy patients (23).

Related to phenolic compounds absorption, the influence of the delivery vehicle seems to be crucial. In human beings, HOTYR absorption was higher when subjects ingested virgin olive oil than in refined oil (phenol-free) enriched with phenolic compounds, or when HOTYR was incorporated into a yogurt as functional food, with urinary recoveries of 44, 23, and 5.8%, respectively (21). In this direction, clinical trials have demonstrated that the amount of hydroxytyrosol in free form was undetectable being 98% in conjugated form, mainly glucuronides, in plasma, and urine (9, 24). These data provided an idea of the bioavailability of hydroxytyrosol, to highlight that olive oil phenolics are well absorbed at the intestinal level, suggesting that the intestinal/hepatic metabolism of the ingested phenolics is extensive. Almost all phenolic content are present in plasma and urine in conjugated forms mainly glucurono-conjugates. Taking into account that the amount of free HOTYR in plasma and urine is almost undetectable, some authors suggest to attribute the biological activity to metabolites of HOTYR. In this direction, there are controversial studies about the biological activities of HOTYR and its conjugated forms. In a study in rats, 3-*O*-glucuronide conjugated HOTYR showed higher activity as a radical scavenger than its corresponding free form (25). More recently, Khymenets et al. (26) showed that two glucuronides, 3-*O*-Gluc-HOTYR and 4-*O*-Gluc-HOTYR, did not show radical scavenging activity and capacity to protect low-density lipoproteins (LDL) against oxidation compare to HOTYR. In both articles, DPPH assay is used but under different concentrations and solvent. Thus, there is a need for standardization for free-radical assays in order to provide reliable data (27).

## Cardiovascular Diseases

Numerous studies have demonstrated the importance of naturally occurring dietary polyphenols in promoting cardiovascular health (28). Within the Mediterranean diet much research has focused on active phenolic compounds present in virgin olive oils to aid in explaining reduced mortality and morbidity observed (29). Among them, two isolated compounds, oleuropein and hydroxytyrosol, have received special attention (30). These compounds show several anti-inflammatory and anti-atherogenic activities, such as the inhibition of LDL oxidation, platelet aggregation, and other factors involved in the development of atherosclerosis.

### Effects on endothelial dysfunction

Reactive oxygen species (ROS) are critically involved in the endothelial dysfunction that contributes to atherosclerosis development. Oxidative stress-induced endothelial dysfunction probably represents one of the first stages in the development of atherosclerotic lesions (31, 32). Accordingly, the atherosclerotic vessel wall contains increased levels of ROS, which affect several redox–sensitive pathways in vascular cells, resulting in a markedly altered cellular composition of the tissue. Migration and proliferation of vascular smooth muscle cells in the area is induced, as well as expression of adhesion molecules and chemotactic factors by the endothelium (33). Consequently, increased arterial adhesiveness at predisposed sites provides an excellent environment for local infiltration of circulating immune cells, resulting in chronic inflammation (34). Thus, direct reduction of levels of ROS and/or stimulation of anti-oxidant defenses at these levels could avoid atherosclerosis development.

It has been observed in animal models that levels of several anti-oxidant enzymes decline during atherogenesis (35). Incubation of porcine pulmonary artery endothelial cells (VECs) with HOTYR in the presence of H2O2 prevents the increase in intracellular ROS levels (36). HOTYR increases catalase mRNA, protein, and activity of both cytosolic and nuclear protein levels of forkhead transcription factor 3a (FOXO3a), which directly increases the expression of anti-oxidant enzymes, as well as the phosphorylation of AMP-activated protein kinase (AMPK) that translocates FOXO3a to the nucleus. Thus, these findings demonstrate that HOTYR positively regulates the anti-oxidant defense system in VECs and provides a molecular basis for the prevention of cardiovascular diseases by HOTYR.

Another study analyzed the effects of HOTYR on anti-oxidant status and the progression of aortic lesions in hyperlipemic rabbits. After 1-month of controlled diet 50 and 42% decrease in total cholesterol and triacylglycerols, respectively, and a 2.3-fold increase in HDL-cholesterol were observed. The HOTYR-supplemented groups also improved their anti-oxidant status and reduced the size of atherosclerotic lesions measured as intimal layer areas of the aortic arch when compared with control animals (37).

In 2003, Carluccio et al. (38) found that at nutritionally relevant concentrations, oleuropein and hydroxytyrosol, among all the tested molecules present in olive oil and red wine, reduced monocytoid cell adhesion to stimulated endothelium, as well as vascular cell adhesion molecule-1 (VCAM-1) mRNA and protein in human umbilical vein endothelial cells incubated for 30 min with the phytochemicals, followed by co-incubation with bacterial lipopolysaccharide or cytokines to trigger adhesion molecule expression. The relevance of nuclear factor-NF-κB and activator protein-1 confirmed by electrophoretic mobility shift assays, and possibly GATA binding sites in mediating VCAM-1 transcriptional inhibition, was observed in this case.

### Protection of LDL particles from oxidative damage

In 1995, an experimental study conducted by Visioli showed *in vitro* the inhibitory effect of hydroxytyrosol and two derivative compounds on LDL oxidation (39). Different indices of lipid oxidation such as vitamin E content, formation of thiobarbituric acid-reacting substances (TBARS), lipid peroxidases, levels of polyunsaturated fatty acids, protein modification, and conjugated diene formation were used. Copper sulfate and horseradish peroxidase/hydrogen peroxidase were used as LDL oxidation systems. Hydroxytyrosol and two derivatives at 10^−5^ M almost completely inhibited conjugated diene formation induced by 5 μM copper sulfate as oxidant in LDL incubation samples. The mechanism of action proposed for this phenolic compounds were the scavenging free radicals and/or acting as chelating agents and thus depressing the superoxide-driven reactions and breaking the propagation chain of peroxides (40).

More than 15 years after the first study, the European Food Safety Authority (EFSA) published a scientific opinion related to polyphenols in olive, and The EFSA Panel on Dietetic Products, Nutrition, and Allergies regarded about the relationship between the consumption of olive oil polyphenols and protection of LDL particles from oxidative damage (41). The panel considered that 5 mg of hydroxytyrosol and its derivatives in olive oil should be consumed daily to reach this effect at physiological level. This amount of hydroxytyrosol must be contained in a maximum of 20 g of oil to be considered within a balanced diet. Considering that the oxidation of LDL plays an important role in the development of atherosclerosis, this communication brings robust data on the implication of phenolic compounds in coronary heart disease. This scientific opinion was supported by consistent studies showing the relationship between phenolic compounds in olive oil and LDL oxidation. To provide its opinion EFSA took into account several studies demonstrating the involvement of phenolic compounds as protectors of LDL oxidation. Other studies have been reviewed with results in the same direction as those previously recognized by EFSA. Data are shown in Table 1.

**Table 1:** Protection of LDL from oxidation

Study type	Anti-oxidant capacity measurement	Conclusion	Reference
Incubation of hydroxytyrosol and two conjugated of HOTYR with isolated LDL	Concentration Vit.E in LDL in presence of copper sulfate with different concentration HOTYR	HOTYR and conjugated, copper sulfate-induced LDL oxidation	(39)
	TBARS level	
	Levels of lipid peroxide	
	Conjugated dienes formation	
Refined olive oil, common olive oil and virgin olive oil were added to isolated low-density lipoprotein	Conjugated dienes formation was monitored after copper-mediated LDL oxidation	Oils with higher content of phenolic compounds showed the greatest protection of LDL oxidation	(42)
Dietary study in human beings consuming virgin olive oil	Variables used to study the resistance of LDL: lag time, maximum amount of dienes and maximal oxidation rate	Decrease in LDL cholesterol and LDL oxidation during one week period consuming 25 ml of olive oil. Significantly levels of MUFA and anti-oxidants in LDL	(43)
Double-blind, crossover, randomized, controlled clinical trial to investigate the effects of olive oil with differences in their phenolic content on LDL oxidation	Ox-LDL was determined in plasma by a sandwich ELISA procedure. The LDL resistance to oxidation was determined by formation of conjugated dienes after copper oxidation of isolated LDL	Decrease *in vivo* LDL oxidation and resistance to *ex vivo* (isolated) LDL oxidation	(44)
Double-blind, randomized, crossover experimental trial in human beings consuming olive oil	Ox-LDL measured by the ELISA method	Significant decrease in plasma oxidized LDL in dose-dependent manner in relation with the phenolic content of the olive oil ingested	(45)
Multicenter controlled human intervention consuming olive oil with differences in their phenolic content (EUROLIVE)	Ox-LDL measured by a sandwich ELISA procedure using murine monoclonal antibody mAb-4E6	Significant decrease in plasma oxidized LDL in dose-dependent manner in relation with the phenolic content of the olive oil ingested	(46)
Dietary study in human beings consuming olive oil with differences in their phenolic content	Ox-LDL measured by enzyme immunoassay using murine monoclonal antibody mAb-4E6	Dose-dependent response between the intake of polyphenols and the decrease in LDL peroxidation	(47)
Subsample of EUROLIVE study. Human intervention consuming olive oil with differences in their phenolic content	Ox-LDL in plasma measured by a sandwich ELISA with murine monoclonal antibody mAb-4E6. Anti-oxidant capacity of biological metabolites of phenolic compounds (hydroxytyrosol monosulphate and homovanillic acid sulfate)	Inverse relationship between these metabolites and the degree of LDL oxidation	(48)

*HOTYR, hydroxytyrosol; LDL, low-density lipoproteins; Vit. E, vitamin E; CuSO4, copper sulfate; TBARS, thiobarbituric acid-reacting substances; ELISA, enzyme-linked immunosorbent assay; MUFA, monounsaturated fatty acids; Ox-LDL, oxidized low-density lipoproteins*.

In general, olive oil polyphenols may have anti-oxidant protective properties not only by the free-radical scavenging but also for the modulation of gene expression such the CD40 ligation and its downstream products in human beings. It has been observed that intake of high content of phenolic compounds from olive oil (366 mg/kg) reduced the expression of CD40 ligand gene and its downstream products, and was associated with a decrease in oxidized LDL in plasma and an increase in urinary polyphenols (40).

### Inhibition of platelet aggregation

Another process involved in the development of atherosclerotic lessions is platelet aggregation, which is the previous step in thrombus formation (49). HOTYR can be considered anti-thrombotic, since it significantly reduces platelet aggregation. While Dell’Agli et al. (50) found positive results for HOTYR in the reduction of human platelet aggregability but not through the inhibition of cAMP- and cGMP-phosphodiesterases as the targets of the biological effect as the other olive oil polyphenols tested in their study, early works of Petroni et al. (51) and more recently Léger et al. (52) in human blood samples from healthy volunteers and patients with uncomplicated diabetes type 1, respectively, also suggest anti-platelet aggregation activity. In these studies, a reduction in thromboxane B2 levels, a chemically stable and inactive form of thromboxane A_2_, which acts as platelet aggregating and vasoconstrictor, was measured. Another comparative study (53) with mildly dyslipidemic patients reached similar results, finding a decrease in the production of thromboxane B2 and an increase in plasma anti-oxidant capacity after consumption of HOTYR contained in extra virgin olive oil (166 mg/l; estimated ingestion 6.64 mg/day) compared with the absence of results in the case of a low intake from a refined oil (2 mg/l; estimated ingestion 0.02 mg/day).

Gonzalez-Correa et al. (54) also found HOTYR and HOTYR-acetate as inhibitor of platelet aggregation in rats after oral administration for 7 days, with similar effectiveness of acetyl salicylic acid. In this case, mechanisms involved in this effect were a decrease in thromboxane synthesis and an increase in vascular nitric oxide (NO) production.

### Anti-inflammatory effects

*In vitro* and *in vivo* studies (55) taken as a model of inflammatory response in rats plantar edema induced by carrageenan shows hydroxytyrosol as inhibitor of cyclooxygenases 1 and 2, which convert arachidonic acid to prostaglandins, resulting in pain and inflammation, at the same level as widely used drugs such as ibuprofen and celecoxib. In studies with human monocytic cells, THP-1 (56) treated with lipopolysaccharides to induce inflammatory response HOTYR showed the inhibition of pro-inflammatory cytokines (TNF-α) and reduced the expression of cyclooxygenase-2 and inducible nitric oxide synthase (iNOS) more than 60%.

Beneficial effects were also found (57) in patients with stabilized coronary disease. The intake of virgin olive oil, rich in polyphenols, was shown to be more effective than refined olive oil in producing a decrease in interleukin-6 (IL-6) and C-reactive protein (CRP) levels, recognized inflammatory markers in cardiovascular disease (58).

A DSM study from the human nutrition and health department (59) describes hydroxytyrosol as the most powerful anti-inflammatory compound among polyphenols of olive oil, showing effective inhibition in the production of NO and PGE2, decreasing secretion of cytokines (IL-1α, IL-1β, IL-6, IL-12, TNF-α) and chemokines (CXCL10/IP-10, CCL2/MPC-1) and reducing gene expression of iNOS, IL-1α, CXCL10/IP-10, MIP-1β, matrix metaloproteinase-9, and prostaglandin E2 synthase (PGES).

Very recently, Killen et al. (60) have reviewed the HOTYR ability to modulate the transcription factor NF-κB whose activation triggers the expression of over 150 genes, including those encoding cytokines, such as tumor necrosis factor alpha (TNF-α), interleukin (IL)-1, IL-6, and IL-17, chemokines and adhesion molecules, implicated in both physiological and pathophysiological inflammatory responses.

Data from these studies provide a molecular basis for elucidating the effects of HT on inflammatory processes. Concentrations reached after dietary intake of olive oil results in appropriated values of HOTYR for inhibiting the NO and PGE2 production and significant modulation of gene expression. Therefore, exposure to lower levels of HOTYR by regular dietary intake might repeatedly modulate physiological parameters that provide a systemic health benefit.

## Hydroxytyrosol and Cancer

Cancer is among the most deadly diseases today. For this reason, scientists have sought alternatives to prevent these diseases or to improve the quality of life of patients. It has been estimated that more than two thirds of human cancers could be prevented through appropriate lifestyle, which includes a proper nutrition (61). Over the past 20 years, many studies in human beings have indicated an inverse correlation between regular fruit and vegetable consumption and development of various types of cancer (62, 63). Currently, one approach with enormous potential is cancer chemoprevention (64). Chemoprevention as first defined by Sporn in 1976, uses natural, synthetic, or biologic chemicals agents to reverse, suppress, or prevent carcinogenic progression to invasive cancer (65). The chemoprevention by dietary phytochemicals is gaining increasing attention because of their ability to modulate a plethora of signal transduction pathways in different types of cancer (66).

Polyphenols are found within the group of substances with chemo-preventive properties as they affect several mechanisms, including removal of cancer agents, modulation of cancer cell signaling and cell cycle progression, promotion of apoptosis, and modulation of enzymatic activities. High concentrations of polyphenols from olive and olive products, throughout history, have been heavily exploited for risk reduction of several chronic diseases. In particular, the consumption of extra virgin olive oil is associated with a risk reduction of certain cancers supported by several epidemiological studies in human beings (67). These health benefits can be attributed more toward phenolic compounds than to its fatty acid profile (68, 69). Highlighting, HOTYR is one of the main phenolic components that has shown potent anti-oxidant, anti-atherogenic, anti-thrombotic, and anti-inflammatory activity (70). In other cases, derivatives like oleuropein and other related polyphenols instead of HOTYR were found to be responsible for cytotoxic activity (71, 72).

Regarding the cancer chemo-preventive activities, it has been shown that HOTYR is able to inhibit both initiation and promotion/progression phases of carcinogenesis by preventing the DNA damage induced by different genotoxic molecules and by inhibiting proliferation and inducing apoptosis in different tumors cell lines, respectively (73, 74). Cell culture studies constitute a valuable tool for identifying the molecular targets modulated by dietary phenolic compounds in cancer cells and for elucidating the molecular pathways involved in the overall disease process (75). Like a considerable number of anti-oxidants, hydroxytyrosol, and oleuropein are reported to produce extracellular hydrogen peroxide under standard culture conditions (76, 77). Factors responsible for this pro-oxidant behavior were identified (78) and several strategies have been developed to correct artifacts (79). Anyway, pro-apoptotic effects were observed in HL60 cells incubated with HOTYR even in conditions not supporting H2O2 accumulation (between 23.8 and 38.0% depending on the media) suggesting that other mechanisms, in addition to the H2O2-releasing activity, could be involved in the pro-apoptotic activity (80). On the other hand, hydrogen peroxide is also known to play a role in cellular signaling (81). Thus, polyphenols might be beneficial due to its contribution to redox homeostasis. A higher decrease in normal levels of ROS could be detrimental to health, which could be also an explanation to negative results obtained in several studies (64, 65).

The role of HOTYR as a chemo-preventive anti-carcinogenic compound has received attention in human carcinoma cells in many *in vitro* studies showing significant effects in several cancer cell lines as main responsible compound, either alone or in a synergistic manner (82), arresting the cell cycle at any of its phases, producing differentiation, apoptosis, or preventing DNA from oxidative stress (Table 2).

**Table 2:** Chemo-preventive properties of hydroxytyrosol in studies of cancer cell lines

Cancer	Cell line	Cellular effects	Capacity of HT in cancer	Reference
Leukemia	HL60	Cell cycle arrest (G0/G1 or G2/M)	Antiproliferative	(74)
			Pro-apoptotic	(83, 84)
		Inhibition on DNA synthesis	Antiproliferative and pro-apoptotic	(85)
		Induction of differentiation	
Colon cancer	HT29	G2/M phase cell cycle arrest	Antiproliferative	(74)
		Growth arrest. Stress of the endoplasmic reticulum. Inhibits NF-κB	Antiproliferative and pro-apoptotic	(86)
		G2/M phase cycle arrest	
	Caco-2	Inhibition of p38/CREB phosphorylation and reduction COX-2 expression	Antiproliferative	(87)
		Inhibition of ERK1/2 phophorilation and cyclin D1 levels	Antiproliferative	(88)
		Up-regulating p21 and CCNG2 and down-regulating CCNB1 protein expression	Pro-apoptotic	(89)
	SW620	S phase cell cycle arrest. Inhibition of FAS expression	Antiproliferative and pro-apoptotic	(90)
	HT115	Inhibits invasion of cancer colon cells	Anti-invasive	(91)
Breast cancer	MCF-7	G0/G1 cell cycle arrest	Antiproleferative and pro-apoptotic	(92, 93)
		Inhibition of estradiol induced ERK1/2 phosphorylation	Antiproliferative	(94)
	SKBR3	Delay cell cycle in G2/M phase	Antiproliferative and pro-apoptotic	(95)
	T-47D	
	MB231	Inhibits CCL5 accumulation and consequently the increase in the ERK1/2-cyclin D1 pro-proliferative pathway	Antiproliferative and pro-apoptotic	(96)
Hepatocellular carcinoma	HCC	Induction of G2/M cell cycle arrest and apoptosis	Antiproliferative and pro-apoptotic	(97)
		Suppression of the AKT and NF-κB pathways activation	
	HepG2	Protection of oxidative stress produced by tBuOOH		(98)
Cholangio-carcinoma	TFK-1	G2/M phase cell cycle arrest	Antiproliferative and pro-apoptotic	(99)
	KMBC	Inhibition of phospho-ERK	
	GBS-SD			

*COX-2, cyclooxygenase-2; DNA, deoxyribonucleic acid; ERK1/2, extracellular-signal-regulated kinases 1 and 2; cyclin D1, cyclin-dependent kinase 1; CCNG2 and CCNB1, genes that encode cyclin G2 (inhibitor or cell cycle progression) and cyclin B1 (regulatory protein evolved in mitosis); FAS, fatty acid synthase; NF-κB, nuclear factor kappa-light-chain-enhancer of activated B cells; p21, cyclin-dependent kinase inhibitor 1A (also known as p21^WAF1/Cip1^); CCL5, chemotactic citokine C–C motif ligand 5; AKT, protein kinase B (involved in metabolism, apoptosis, and proliferation processes); tBuOOH, tert-butyl hydroperoxide; ERK, extracellular signal-regulated kinases*.

Besides, some structural modifications trying to incorporate the well-known benefits of organo-sulfur compounds have been also done. Recently, it was found that the introduction of sulfur containing functional groups on HOTYR molecule leads to an enhancement of chemo-preventive activity in HL60 and HL60R cell lines, suggesting that they could be able to reverse the resistance toward the most common anti-cancer drugs (100).

Briefly summarizing, the general approach to health benefits of polyphenols comes from their anti-oxidant and anti-inflammatory effects (101). These effects can be observed in *in vivo* studies with HOTYR. As an example, it showed protection against oxidative stress induced by Cyclosporine-A by scavenging most radical species, measured by the increase of GSH/GSSG ratio and the decrease of lipid peroxidation levels in tubular cells in rats (102). Another study that looked at anti-cancer effects of HOTYR is found in the inhibition of growth and cell proliferation in Sprague Dawley rats with experimental mammary tumors (103). In addition to these findings, another *in vivo* study with HOTYR (250 and 500 mg/kg/day) markedly inhibited the growth of cholangio-carcinoma xenografts in mice, through G2/M phase cell cycle arrest and apoptosis (99).

Epidemiological studies have suggested that HOTYR may have protective effects against several types of cancer (104). Likewise, antitumor effects of olive derivatives have been reported for different organs of the body such as the pancreas, oral cavity, esophagus, colon–rectum, prostate, and lung (105). In the colon cancer case it is mentioned that if the HOTYR would reach the colon in a distribution volume of around 250 ml, it would result in a concentration of approximately 72 μmol/l, a value that induced a 50% inhibition of cell proliferation. Accordingly may exert chemo-preventive activity on colon cancer inhibiting cell proliferation (106).

As shown, most studies to date have been investigated in cell lines and a relative few number of studies are in animals or human beings. Therefore, for a better understanding of the potential and mechanisms of HOTYR specific antitumor activity, both *in vitro* and *in vivo*, and with animals and human beings studies are necessary.

## Effects of HOTYR on Human Immunodefiency Virus

Acquired immunodeficiency syndrome (AIDS) is a disease of the human immune system caused by infection with human immunodeficiency virus (HIV).

Despite the existence of drugs and combinations against the HIV virus to reduce the morbidity and mortality of patients, they do not cure the infection. Moreover, anti-HIV therapies must be maintained long-term with important chronic toxicity and drug resistance problems. Therefore, further research is needed to reach the complete healing. Meanwhile, the prevention of transmission and virus proliferation, as well as palliatives against HIV-derived diseases plays a crucial role. At the final of the last century up to half of patients infected with the HIV virus were estimated to use alternative therapies in addition to standard therapy (107). As an example, some efforts were made to diminish side effects of this disease such as oral thrush or diarrhea, with alternative therapies such as lemon juice or lemon grass infusion (108) or traditional Chinese herbal medicines (109).

In 2003, Lee-Huang et al. (110), in an *in vitro* study observed that OLE inhibits acute infection and cell-to-cell transmission between uninfected MT2 cells co-cultured with human immunodeficiency virus type 1 (HIV-1) infected H9 T lymphocytes in a dose-dependent manner, with an EC_50_ of around 0.2 μg/ml. Moreover, OLE also inhibits HIV-1 replication, as assayed by p24 (capside virus protein) expression an EC_50_ of 0.23 μg/ml. It was found that oleuropein, the derivative of HOTYR, was the OLE compound responsible of the highest anti-HIV activity.

In this sense, newly studies using molecular docking simulation showed that oleuropein and HOTYR were the effective components of OLE, which inhibit the HIV-1 fusion core formation and it was confirmed by EC50s of 66–58 nM. They are active against multiple stages of HIV-1 life cycle, inhibiting cell-to-cell transmission and viral core antigen p24 production. In one hand, in HIV-1 fusion, the transmembrane subunit gp41 from HIV-1 envelope glycoprotein mediates fusion of the virus with the target cells and both oleuropein and HOTYR are also able to inhibit the fusion promoting refolding of gp41. This study compared oleuropein and HOTYR with Furzeon (T-20 or Enfuvirtide), the only HIV fusion inhibitor approved by the FDA, in terms of fabrication given the difficulty of manufacturing of this drug due to this high-molecular weight. Instead of this oleuropein and HOTYR are easily obtainable from olive oil or OLE and also can be synthesized in the laboratory (111). On the other hand, the same authors examined the molecular integration of OLE and HOTYR with HIV-1 integrase, which is an essential enzyme for retroviral replication and is involved in the integration of HIV DNA into host chromosomal DNA. They observed that OLE and HOTYR exhibited dose-dependent inhibition on all three activities of this enzyme including 3′-processing, strand transfer, and disintegration (112).

The use of natural small molecules, combining anti-inflammatory and anti-viral effects has recently received special attention to prevent HIV infection and even to palliate HIV-derived diseases. In the case of HOTYR, this has been reflected in two patents in the last decade, either for reducing inflammatory derived diseases (113) or for direct prevention of the HIV infection (114).

## Current and Potential Uses in Health Care

We have reviewed the properties of hydroxytyrosol, focusing on the protection against cardiovascular diseases and progresses on diseases like cancer or AIDS. Other benefits can be attributed to this and other phenolic compounds in olive such as maintenance of normal blood HDL-cholesterol concentrations, maintenance of normal blood pressure, anti-inflammatory properties, contribution to the upper respiratory tract health, maintenance a normal function of gastrointestinal tract, and contribution to body defenses against external agents, although only a cause-effect relationship between the consumption of olive oil polyphenols and protection of LDL particles from oxidative damage has been firmly recognized (41).

More conclusive studies are needed to link all these potential benefits with phenolic compounds such as HOTYR and OLE and thus to involve new lines of research into drugs that prevent and treat these diseases. On the other hand, according to other reviews (41, 115), the average intake of olive oil ranges 30–50 g/day in the Mediterranean countries. This would be an estimated intake around 9 mg of phenolic compounds per day. In a recent study, De la Torre et al. (116) estimate the intake of tyrosol and HOTYR from virgin olive oil of a population from southern Spain in the range of 88.5–237.4 μg/day. Another estimation based on a study of Greek origin with six types of olive oil, which analyzes the concentration of phenolic compounds, tyrosol, and HOTYR, concluded that the daily intake of hydroxytyrosol in the Mediterranean area would be 2 mg (considering the maximum 50 mg/day) (115). This amount would be insufficient to reach the recommended amount of 5 mg to develop the benefit of protection of LDL particles from oxidative damage. It would be possible to consider supplements or nutraceutics that may ensure that doses. In fact, different products are already in the market containing hydroxytyrosol as Mediteanox^®^, Hydrox^®^, and Hytolive^®^. Different types of presentations appearing on the market, such as capsules, elixir, creams, and even an extra virgin olive oil with a very high content of hydroxytyrosol (over 500 mg/kg). Hydrox^®^ and Hytolive^®^ have been granted the generally recognized as safe (GRAS). Even synthetic pure hydroxytyrosol, commercialized by SEPROX BIOTECH, has reached this status and it has been recently proposed in EU for Novel Food. Some of these products have been already tested successfully showing some of the previously described health benefits (117–119).

The lack of adverse effects at high levels has been pointed out several times at acute dose (120) or after prolonged consumption (121). Pure hydroxytyrosol has been also evaluated in a recent toxicological study in rats, and a no observed adverse effects level (NOAEL) of 500 mg/kg/day of hydroxytyrosol has been proposed. The toxicological evaluation included clinical signs, grip strength, locomotor activity, food consumption, body weight, hematology, clinical biochemistry, pathology, organ weights, and microscopic findings and the authors did not find toxicological relevance effects (122).

Besides, the European Commission has funded a research that will try to test the efficacy of hydroxytyrosol in the prevention of HIV infection. In a few years, this compound could become a microbicide gel to prevent HIV transmission during sex (123).

We may conclude that HOTYR is an extraordinary molecule with recognized beneficial properties. Some of them are well-contrasted and recognized by safety agencies. However, further investigations are necessary for better understanding of its mechanism of action on other diseases such as cancers or AIDS. This will clarify important characteristics of this substance, which helps in the prevention for these diseases.

## Conflict of Interest Statement

The authors declare that the research was conducted in the absence of any commercial or financial relationships that could be construed as a potential conflict of interest.

## References

[1] Genevieve Buckland CAG Trends in olive production, supply and consumption in Mediterranean countries from 1961 to the present day. In: Inc E, editor. Olives and Olive Oil in Health and Disease Prevention (2010). p. 689–98.

[2] Kromhout D Keys A Aravanis C Buzina R Fidanza F Giampaoli S Food consumption patterns in the 1960s in seven countries. Am J Clin Nutr (1989) 49:889–94. 2718924 10.1093/ajcn/49.5.889

[3] FDA Allows Qualified Health Claim to Decrease Risk of Coronary Heart Disease. FDA News Release (2004). Available from: http://www.fda.gov/newsevents/newsroom/pressannouncements/2004/ucm108368.htm

[4] Boskou D Olive Oil. Chemistry and Technology. Second ed Champaign, IL: AOCS Press (2006).

[5] Soler-Rivas C Espin JC Wichers HJ Oleuropein and related compounds. J Sci Food Agric (2000) 80(7):1013–23 10.1002/(SICI)1097-0010(20000515)80:7<1013::AID-JSFA571>3.0.CO;2-C

[6] Owen RW Mier W Giacosa A Hull WE Spiegelhalder B Bartsch H. Phenolic compounds and squalene in olive oils: the concentration and antioxidant potential of total phenols, simple phenols, secoiridoids, lignans and squalene. Food Chem Toxicol (2000) 38(8):647–59. 10.1016/S0278-6915(00)00061-2 10908812

[7] Romero C Brenes M Yousfi K García P García A Garrido A. Effect of cultivar and processing method on the contents of polyphenols in table olives. J Agric Food Chem (2004) 52:479–84. 10.1021/jf030525l 14759136

[8] Visioli F Bellomo G Galli C. Free radical-scavenging properties of olive oil polyphenols. Biochem Biophys Res Commun (1998) 247(1):60–4. 10.1006/bbrc.1998.8735 9636654

[9] Visioli F Galli C Bornet F Mattei A Patelli R Galli G Olive oil phenolics are dose-dependently absorbed in humans. FEBS Lett (2000) 468(2–3):159–60. 10.1016/S0014-5793(00)01216-3 10692578

[10] Boskou D Olive Oil: Minor Constituents and Health. Boca Raton, FL: CRC Press (2008).

[11] Esti M Cinquanta L La Notte E. Phenolic compounds in different olive varieties. J Agric Food Chem (1998) 46(1):32–5. 10.1021/jf970391+ 10554192

[12] Charoenprasert S Mitchell A. Factors influencing phenolic compounds in table olives (*Olea europaea*). J Agric Food Chem (2012) 60:7081–95. 10.1021/jf3017699 22720792

[13] Owen RW Haubner R Mier W Giacosa A Hull WE Spiegelhalder B Isolation, structure elucidation and antioxidant potential of the major phenolic and flavonoid compounds in brined olive drupes. Food Chem Toxicol (2003) 41:703–17. 10.1016/S0278-6915(03)00011-5 12659724

[14] Dimitrios B Sources of natural phenolic antioxidants. Trends Food Sci Technol (2006) 17(9):505–12 10.1016/j.tifs.2006.04.004

[15] Fernandez-Bolaños J Heredia A Felizon B Brenes M Guillen R Rodriguez R Procedimiento de obtencion de hidroxitirosol a partir de hueso de aceituna. ES 2145701 B1 (2001).

[16] Fernández-Mar MI Mateos R García-Parrilla MC Puertas B Cantos-Villar E Bioactive compounds in wine: resveratrol, hydroxytyrosol and melatonin: a review. Food Chem (2012) 130(4):797–813 10.1016/j.foodchem.2011.08.023

[17] de la Torre R Covas M Pujadas M Fitó M Farré M. Is dopamine behind the health benefits of red wine? Eur J Nutr (2006) 45(5):307–10. 10.1007/s00394-006-0596-9 16586149

[18] Bai C Yan X Takenaka M Sekiya K Nagata T Determination of synthetic hydroxytyrosol in rat plasma by GC-MS. J Agric Food Chem (1998) 46(10):3998–4001 10.1021/jf980451r

[19] Tuck KL Freeman MP Hayball PJ Stretch GL Stupans I. The in vivo fate of hydroxytyrosol and tyrosol, antioxidant phenolic constituents of olive oil, after intravenous and oral dosing of labeled compounds to rats. J Nutr (2001) 131(7):1993–6. 11435519 10.1093/jn/131.7.1993

[20] Miro-Casas E Covas MI Fito M Farre-Albadalejo M Marrugat J de la Torre R. Tyrosol and hydroxytyrosol are absorbed from moderate and sustained doses of virgin olive oil in humans. Eur J Clin Nutr (2003) 57(1):186–90. 10.1038/sj.ejcn.1601532 12548315

[21] Visioli F Galli C Grande S Colonnelli K Patelli C Galli G Hydroxytyrosol excretion differs between rats and humans and depends on the vehicle of administration. J Nutr (2003) 133(8):2612–5. 12888646 10.1093/jn/133.8.2612

[22] De la Torre R. Bioavailability of olive oil phenolic compounds in humans. Inflammopharmacology (2008) 16:245–7. 10.1007/s10787-008-8029-4 18815736

[23] Vissers MN Zock PL Roodenburg AJC Leenen R Katan MB. Olive oil phenols are absorbed in humans. J Nutr (2002) 132:409–17. 11880564 10.1093/jn/132.3.409

[24] Miro-Casas E Covas MI Farre M Fito M Ortuño J Weinbrenner T Hydroxytyrosol disposition in humans. Clin Chem (2003) 49(6):945–52 10.1373/49.6.945 12765992

[25] Tuck KL Hayball PJ Stupans I. Structural characterization of the metabolites of hydroxytyrosol, the principal phenolic component in olive oil, in rats. J Agric Food Chem (2002) 50(8):2404–9. 10.1021/jf011264n 11929304

[26] Khymenets O Fitó M Touriño S Muñoz-Aguayo D Pujadas M Torres JL Antioxidant activities of hydroxytyrosol main metabolites do not contribute to beneficial health effects after olive oil ingestion. Drug Metab Dispos (2010) 38(9):1417–21. 10.1124/dmd.110.032821 20516254

[27] Prior RR Wu X Schaich K. Standardized methods for the determination of antioxidant capacity and phenolics in foods and dietary supplements. J Agric Food Chem (2005) 53:4290–302. 10.1021/jf0502698 15884874

[28] Khurana S Venkataraman K Hollingworth A Piche M Tai TC. Polyphenols: benefits to the cardiovascular system in health and in aging. Nutrients (2013) 5:3779–827. 10.3390/nu5103779 24077237 PMC3820045

[29] Cicerale S Lucas L Keast R. Biological activities of phenolic compounds present in virgin olive oil. Int J Mol Sci (2010) 11:458–79. 10.3390/ijms11020458 20386648 PMC2852848

[30] Bulotta S Celano M Lepore SM Montalcini T Pujia A Russo D. Beneficial effects of the olive oil phenolic components oleuropein and hydroxytyrosol: focus on protection against cardiovascular and metabolic diseases. J Transl Med (2014) 12(1):219. 10.1186/s12967-014-0219-9 25086598 PMC4237885

[31] Dhalla NS Temsah RM Netticadan T Role of oxidative stress in cardiovascular diseases. J Hypertens (2000) 18(6):655–73 10.1097/00004872-200018060-00002 10872549

[32] Witztum JL Steinberg D. Role of oxidized low density lipoprotein in atherogenesis. J Clin Invest (1991) 88:1785–92. 10.1172/JCI115499 1752940 PMC295745

[33] De Nigris F Lerman LO Condorelli M Lerman A Napoli C. Oxidation-sensitive transcription factors and molecular mechanisms in the arterial wall. Antioxid Redox Signal (2001) 3(6):1119–30. 10.1089/152308601317203620 11813985

[34] Spagnoli LG Bonanno E Sangiorgi G Mauriello A Role of inflammation in atherosclerosis. J Nucl Med (2007) 48(11):1800–15 10.2967/jnumed.107.038661 17942804

[35] ’t Hoen PAC Van der Lans CAC Van Eck M Bijsterbosch MK Van Berkel TJC Twisk J Aorta of ApoE-deficient mice responds to atherogenic stimuli by a prelesional increase and ubsequent decrease in the expression of antioxidant enzymes. Circulation Research (2003) 93:262–9. 12829615 10.1161/01.RES.0000082978.92494.B1

[36] Zrelli H Mieko Matsuoka M Kitazaki S Zarrouk M Miyazaki H. Hydroxytyrosol reduces intracellular reactive oxygen species levels in vascular endothelial cells by upregulating catalase expression through the AMPK-FOXO3a pathway. Eur J Pharmacol (2011) 660:275–82. 10.1016/j.ejphar.2011.03.045 21497591

[37] Gonzalez-Santiago M Martín-Bautista E Carrero JJ Fonollá J Baró L Bartolomé MV One-month administration of hydroxytyrosol, a phenolic antioxidant present in olive oil, to hyperlipidemic rabbits improves blood lipid profile, antioxidant status and reduces atherosclerosis development. Atheroslerosis (2006) 188:35–42 10.1016/j.atherosclerosis.2005.10.022 16300770

[38] Carluccio MA Siculella L Ancora MA Massaro M Scoditti E Storelli C Olive oil and red wine antioxidant polyphenols inhibit endothelial activation. Antiatherogenic properties of Mediterranean diet phytochemicals. Arterioscler Thromb Vasc Biol (2003) 23:622–9. 10.1161/01.ATV.0000062884.69432.A0 12615669

[39] Visioli F Bellomo G Montedoro G Galli C. Low density lipoprotein oxidation is inhibited in vitro by olive oil constituents. Atherosclerosis (1995) 117(1):25–32. 10.1016/0021-9150(95)05546-9 8546752

[40] Castañer O Covas MI Khymenets O Nyyssonen K Konstantinidou V Zunft HF Protection of LDL from oxidation by olive oil polyphenols is associated with a downregulation of CD40-ligand expression and its downstream products in vivo in humans. Am J Clin Nutr (2012) 95(5):1238–44. 10.3945/ajcn.111.029207 22440854

[41] EFSA Panel on Dietetic Products, Nutrition and Allergies (NDA). Scientific opinion on the substantiation of health claims related to polyphenols in olive and protection of LDL particles from oxidative damage (ID 1333, 1638, 1639, 1696, 2865), maintenance of normal blood HDL-cholesterol concentrations (ID 1639), maintenance of normal blood pressure (ID 3781), “anti-inflammatory properties” (ID 1882), “contributes to the upper respiratory tract health” (ID 3468), “can help to maintain a normal function of gastrointestinal tract” (3779), and “contributes to body defences against external agents” (ID 3467) pursuant to Article 13(1) of Regulation (EC) No 1924/20061. EFSA J (2011) 9(4):2033 10.2903/j.efsa.2011.2033

[42] Fitó M Covas MI Lamuela-Raventós RM Vila J de la Torre C Marrugat J. Aceite de oliva e inhibición de la oxidación de las lipoproteínas de baja densidad. Importancia de los compuestos fenólicos. Med Clín (2000) 115(5):166–9. 10.1016/S0025-7753(00)71497-7 10996871

[43] Gimeno E Fitó M Lamuela-Raventós RM Castellote AI Covas M Farré M Effect of ingestion of virgin olive oil on human low-density lipoprotein composition. Eur J Clin Nutr (2002) 56(2):114–20. 10.1038/sj.ejcn.1601293 11857044

[44] Marrugat J Covas MI Fitó M Schröder H Miró-Casas E Gimeno E Effects of differing phenolic content in dietary olive oil on lipids and LDL oxidation: a randomized controlled trial. Eur J Nutr (2004) 43(3):140–7. 10.1007/s00394-004-0452-8 15168036

[45] Weinbrenner T Fitó M Farré-Albadalejo M Saez GT Rijken P Tormos C Bioavailability of phenolic compounds from olive oil and oxidative/antioxidant status at postprandial state in healthy humans. Drugs Exp Clin Res (2004) 30:207–12. 15700748

[46] Covas MI de la Torre K Farré-Albaladejo M Kaikkonen J Fitó M López-Sabater C Postprandial LDL phenolic content and LDL oxidation are modulated by olive oil phenolic compounds in humans. Free Radic Biol Med (2006) 40(4):608–16. 10.1016/j.freeradbiomed.2005.09.027 16458191

[47] Covas MI Nyyssönen K Poulsen HE Kaikkonen J Zunft HJF Kiesewetter H The effect of polyphenols in olive oil on heart disease risk factors: a randomized trial. Ann Intern Med (2006) 145(5):333–41. 10.7326/0003-4819-145-5-200609050-00006 16954359

[48] de la Torre-Carbot K Chávez-Servín JL Jaúregui O Castellote AI Lamuela-Raventós RM Nurmi T Elevated circulating LDL phenol levels in men who consumed virgin rather than refined olive oil are associated with less oxidation of plasma LDL. J Nutr (2010) 140(3):501–8. 10.3945/jn.109.112912 20089783

[49] Libby P Paul M RidkerP M Hansson GK. Progress and challenges in translating the biology of aterosclerosis. Nature (2011) 473:317–25. 10.1038/nature10146 21593864

[50] Dell’Agli M Mashi O Galli GV Fagnani R Dal Cero E Caruso D Inhibition of platelet aggregation by olive oil phenols via cAMP-phosphodiesterase. Br J Nutr (2008) 99(5):945–51. 10.1017/S0007114507837470 17927845

[51] Petroni A Blasevich M Salami M Papini N Montedoro GF Galli C. Inhibition of platelet aggregation and eicosanoid production by phenolic components of olive oil. Thromb Res (1995) 78(2):151–60. 10.1016/0049-3848(95)00043-7 7482432

[52] Léger CL Carbonneau MA Michel F Mas E Monnier L Cristol JP A thromboxane effect of a hydroxytyrosol-rich oliveoil wastewater extract in patients with uncomplicated type I diabetes. Eur J Clin Nutr (2005) 59:727–30. 10.1038/sj.ejcn.1602133 15798774

[53] Visioli F Caruso D Grande S Bosisio R Villa M Galli G Virgin Olive Oil Study (VOLOS): vasoprotective potential of extra virgin olive oil in mildly dyslipidemic patients. Eur J Nutr (2005) 44:121–7. 10.1007/s00394-004-0504-0 15309433

[54] González-Correa JA Navas MD Muñoz-Marín J Trujillo M Fernández-Bolaños J De La Cruz JP. Effects of hydroxytyrosol and hydroxytyrosol acetate administration to rats on platelet function compared to acetylsalicylic acid. J Agric Food Chem (2008) 56:7872–6. 10.1021/jf801502z 18707113

[55] Procopio A Alcaro S Nardi M Oliverio M Ortuso F Sacchetta P Synthesis, biological evaluation and molecular modelling of oleuropein and its semisynthetic derivatives as cyclooxygenase inhibitors. J Agric Food Chem (2009) 57:11161–7. 10.1021/jf9033305 19908866

[56] Zhang X Cao J Jiang L Zhong L. Suppressive effects of hydroxytyrosol on oxidative stress and nuclear factor-kappaB activation in THP-1 cells. Biol Pharm Bull (2009) 32:578–82. 10.1248/bpb.32.578 19336887

[57] Fitó M Cladellas M de la Torre R Martí J Muñoz D Schröder H Anti-inflammatory effect of virgin olive oil in stable coronary disease patients: a randomized, crossover, controlled trial. Eur J Clin Nutr (2008) 62(4):570–4. 10.1038/sj.ejcn.1602724 17375118

[58] Tzoulaki I Murray GD Lee AJ Rumley A Lowe GDO Fowkes FGR. C-reactive protein, interleukin-6, and soluble adhesion molecules as predictors of progressive peripheral atherosclerosis in the general population. Circulation (2005) 112:976–83. 10.1161/CIRCULATIONAHA.104.513085 16087797

[59] Richard N Arnold S Hoeller U Kilpert C Wertz K Schwager J. Hydroxytyrosol is the major anti-inflammatory compound in aqueous olive extracts and impairs cytokine and chemokine production in macrophages. Planta Med (2011) 77(17):1890–7. 10.1055/s-0031-1280022 21830187

[60] Killeen MJ Linder M Pontoniere P Crea R. NF-kb signaling and chronic inflammatory diseases: exploring the potential of natural products to drive new therapeutic opportunities. Drug Discov Today (2014) 19(4):373–8. 10.1016/j.drudis.2013.11.002 24246683

[61] Umar A Dunn BK Greenwald P Future directions in cancer prevention. Nat Rev Cancer (2012) 12(12):835–48 10.1038/nrc3397 23151603

[62] Vauzour D Rodriguez-Mateos A Corona G Oruna-Concha MJ Spencer JP. Polyphenols and human health: prevention of disease and mechanisms of action. Nutrients (2010) 2:1106–31. 10.3390/nu2111106 22254000 PMC3257622

[63] Psaltopoulou T Kosti RI Haidopoulos D Dimopoulos M Panagiotakos DB. Olive oil intake is inversely related to cancer prevalence: a systematic review and a meta-analysis of 13,800 patients and 23,340 controls in 19 observational studies. Lipids Health Dis (2011) 10:127. 10.1186/1476-511X-10-127 21801436 PMC3199852

[64] Steward WP Brown K Cancer chemoprevention: a rapidly evolving field. Br J Cancer (2013) 109(1):1–7 10.1038/bjc.2013.280 23736035 PMC3708589

[65] Tsao AS Kim ES Hong WK Chemoprevention of cancer. CA Cancer J Clin (2004) 54(3):150–80. 15195789 10.3322/canjclin.54.3.150

[66] Priyadarsini RV Nagini S. Cancer chemoprevention by dietary phytochemicals: promises and pitfalls. Curr Pharm Biotechnol (2012) 13(1):125–36. 10.2174/138920112798868610 21466433

[67] Filik L Ozyilkan O Olive-oil consumption and cancer risk. Eur J Clin Nutr (2003) 57:191 10.1038/sj.ejcn.1601497 12548316

[68] Visioli F. Olive oil phenolics: where do we stand? Where should we go? J Sci Food Agric (2012) 92:2017–9. 10.1002/jsfa.5715 22549365

[69] Frankel EN. Nutritional and biological properties of extra virgin olive oil. J Agric Food Chem (2011) 59(3):785–92. 10.1021/jf103813t 21210703

[70] Granados-Principal S Quiles JL Ramirez-Tortosa CL Sanchez-Rovira P Ramirez-Tortosa MC. Hydroxytyrosol: from laboratory investigations to future clinical trials. Nutr Rev (2010) 68(4):191–206. 10.1111/j.1753-4887.2010.00278.x 20416016

[71] Quirantes-Piné R Zurek G Barrajón-Catalán E Bäßmann C Micol V Segura-Carretero A A metabolite-profiling approach to assess the uptake and metabolism of phenolic compounds from olive leaves in SKBR3 cells by HPLC–ESI-QTOF-MS. J Pharm Biomed Anal (2013) 72(0):121–6. 10.1016/j.jpba.2012.09.029 23146235

[72] Menendez JA Vazquez-Martin A Colomer R Brunet J Carrasco-Pancorbo A Garcia-Villalba R Olive oil’s bitter principle reverses acquired autoresistance to transtuzumab (Herceptin^TM^) in HER-overexpressing breast cancer cells. BMC Cancer (2007) 7:80. 10.1186/1471-2407-7-80 17490486 PMC1878493

[73] Fabiani R Rosignoli P De Bartolomeo A Fuccelli R Servili M Montedoro GF Oxidative DNA damage is prevented by extracts of olive oil, hydroxytyrosol, and other olive phenolic compounds in human blood mononuclear cells and HL60 cells. J Nutr (2008) 138(8):1411–6. 18641183 10.1093/jn/138.8.1411

[74] Fabiani RDBA Rosignoli P Servili M Montedoro GF Morozzi G. Cancer chemoprevention by hydroxytyrosol isolated from virgin olive oil through G1 cell cycle arrest and apoptosis. Eur J Cancer Prev (2002) 11(4):351–8. 10.1097/00008469-200208000-00006 12195161

[75] Ramos S. Cancer chemoprevention and chemotherapy: dietary polyphenols and signalling pathways. Mol Nutr Food Res (2008) 52(5):507–26. 10.1002/mnfr.200700326 18435439

[76] Halliwell B. Are polyphenols antioxidants or pro-oxidants? What do we learn from cell culture and in vivo studies? Arch Biochem Biophys (2008) 476:107–12. 10.1016/j.abb.2008.01.028 18284912

[77] Hua Long L Hoi A Halliwell B. Instability of, and generation of hydrogen peroxide by, phenolic compounds in cell culture media. Arch Biochem Biophys (2010) 501(1):162–9. 10.1016/j.abb.2010.06.012 20558131

[78] Odiatou EM Skaltsounis AL Constantinou AI. Identification of the factors responsible for the in vitro pro-oxidant and cytotoxic activities of the olive polyphenols oleuropein and hydroxytyrosol. Cancer Lett (2013) 330:113–21. 10.1016/j.canlet.2012.11.035 23201137

[79] Babich H Schuck AG Weisburg JH Zuckerbraun HL. Research strategies in the study of the pro-oxidant nature of polyphenol nutraceuticals. J Toxicol (2011) 2011:467305. 10.1155/2011/467305 21776260 PMC3135211

[80] Fabiani R Sepporta MV Rosignolli P De Bartolomeo A Crescimanno M Morozzi G. Anti-proliferative and pro-apoptotic activities of hydroxytyrosol on different tumour cells: the role of extracellular production of hydrogen peroxide. Eur J Nutr (2012) 51:455–64. 10.1007/s00394-011-0230-3 21805082

[81] Veal EA Day AM Morgan BA Hydrogen peroxide sensing and signaling. Mol Cell (2007) 26(1):1–14 10.1016/j.molcel.2007.03.016 17434122

[82] Fabiani R Sepporta MV Mazza T Rosignoli P Fuccelli R De Bartolomeo A Influence of cultivar and concentration of selected phenolic constituents on the in vitro chemiopreventive potential of olive oil extracts. J Agric Food Chem (2011) 59:8167–74. 10.1021/jf201459u 21702505

[83] Ragione FD Cucciolla V Borriello A Pietra VD Pontoni G Racioppi L Hydroxytyrosol, a natural molecule occurring in olive oil, induces cytochrome c-dependent apoptosis. Biochem Biophys Res Commun (2000) 278(3):733–9. 10.1006/bbrc.2000.3875 11095977

[84] Fulvio DR Cucciolla V Criniti V Indaco S Borriello A Zappia V. Antioxidants induce different phenotypes by a distinct modulation of signal transduction. FEBS Lett (2002) 532:289–94. 10.1016/S0014-5793(02)03683-9 12482581

[85] Fabiani R Rosignoli P De Bartolomeo A Fuccelli R Morozzi G. Inhibition of cell cycle progression by hydroxytyrosol is associated with upregulation of cyclin-dependent protein kinase inhibitors p21^WAF1/Cip1^ and p27^Kip1^ and with induction of differentiation in HL60 Cells. J Nutr (2008) 138:42–8. 18156402 10.1093/jn/138.1.42

[86] Guichard C Pedruzzi E Fay M Marie JC Braut-Boucher F Daniel F Dihydroxyphenylethanol induces apoptosis by activating serine/threonine protein phosphatase PP2A and promotes the endoplasmic reticulum stress response in human colon carcinoma cells. Carcinogenesis (2006) 27(9):1812–27. 10.1093/carcin/bgl009 16524888

[87] Corona G Deiana M Incani A Vauzour D Dessí MA Spencer JPE. Hydroxytyrosol inhibits the proliferation of human colon adenocarcinoma cells through inhibition of ERK1/2 and cyclin D1. Mol Nutr Food Res (2009) 53(7):897–903. 10.1002/mnfr.200800269 19685549

[88] Corona G Deiana M Incani A Vanzour D Dessi MA Spencer JPE. Inhibition of p38/CREB phosphorylation and COX-2 expression by olive oil polyphenols underlies their anti-proliferative effects. Biochem Biophys Res Commun (2007) 362:606–11. 10.1016/j.bbrc.2007.08.049 17727817

[89] Pereira-Caro G Mateos R Traka MH Bacon JR Bongaerts R Sarriá B Hydroxytyrosyl ethyl ether exhibits stronger intestinal anticarcinogenic potency and effects on transcript profiles compared to hydroxytyrosol. Food Chem (2013) 138:1172–82. 10.1016/j.foodchem.2012.11.118 23411228

[90] Notarnicola M Pisanti S Tutino V Bocale D Rotelli MT Gentile A Effects of olive oil polyphenols on fatty acid synthase gene expression and activity in human colorectal cancer cells. Genes Nutr (2011) 6(1):63–9. 10.1007/s12263-010-0177-7 21437031 PMC3040798

[91] Yumi ZH-Y Hashim IRR McGlynn H Servili M Selvaggini R Taticchi A-SE Inhibitory effects of olive oil phenolics on invasion in human colon adenocarcinoma cells in vitro. Int J Cancer (2008) 122(3):495–500. 10.1002/ijc.23148 17943720

[92] Bouallagui Z Han J Isoda H Sayadi S. Hydroxytyrosol rich extract from olive leaves modulates cell cycle progression in MCF-7 human breast cancer cells. Food Chem Toxicol (2011) 49(1):179–84. 10.1016/j.fct.2010.10.014 20955751

[93] Han J Talorete TP Yamada P Isoda H. Anti-proliferative and apoptotic effects of oleuropein and hydroxytyrosol on human breast cancer MCF-7 cells. Cytotechnology (2009) 59:45–53. 10.1007/s10616-009-9191-2 19353300 PMC2677148

[94] Sirianni R Chimento A De Luca A Casaburi I Rizza P Onofrio A Oleuropein and hydroxytyrosol inhibit MCF-7 breast cancer cell proliferation interfering with ERK1/2 activation. Mol Nutr Food Res (2010) 54:833–40. 10.1002/mnfr.200900111 20013881

[95] Elamin MH Hassan ZK Omer SA Daghestani MH Al-Olayan EM Virk P Apoptotic and antiproliferative activity of olive oil hydroxytyrosol on breast cancer cells. J Med Plants Res (2013) 7(32):2420–8 10.5897/JMPR12.1321

[96] Sarsour EH Goswami M Kalen AL Lafin JT Goswami PC. Hydroxytyrosol inhibits chemokine C-C motif ligand 5 mediated aged quiescent fibroblast-induced stimulation of breast cancer cell proliferation. Age (2014) 36(3):9645. 10.1007/s11357-014-9645-0 24691968 PMC4082566

[97] Zhao B Ma Y Xu Z Wang J Wang F Wang D Hydroxytyrosol, a natural molecule from olive oil, suppresses the growth of human hepatocellular carcinoma cells via inactivating AKT and nuclear factor-kappa B pathways. Cancer Lett (2014) 347(1):79–87. 10.1016/j.canlet.2014.01.028 24486741

[98] Pereira Caro G Mateos R Sarria B Cert R Goya L Bravo L Hydroxytyrosol acetate contributes to the protective effects against oxidative stress of virgin olive oil. Food Chem (2012) 131(3):86978 10.1016/j.foodchem.2011.09.068

[99] Li S Han Z Ma Y Song R Pei T Zheng T Hydroxytyrosol inhibits cholangiocarcinoma tumor growth: an in vivo and in vitro study. Oncol Rep (2014) 31(1):145–52. 10.3892/or.2013.2853 24247752

[100] Sepporta MV López-García MÁ Fabiani R Maya I Fernández-Bolaños JG. Enhanced chemopreventive activity of hydroxytyrosol on HL60 and HL60R cells by chemical conversion into thio derivatives. Eur J Pharm Sci (2013) 48(4–5):790–8. 10.1016/j.ejps.2012.12.028 23313620

[101] Kang NJ Shin SH Lee HJ Lee KW. Polyphenols as small molecular inhibitors of signaling cascades in carcinogenesis. Pharmacol Ther (2011) 130(3):310–24. 10.1016/j.pharmthera.2011.02.004 21356239

[102] Capasso G Di Gennaro CI Della Ragione F Manna C Ciarcia R Florio S In vivo effect of the natural antioxidant hydroxytyrosol on cyclosporine nephrotoxicity in rats. Nephrol Dial Transplant (2008) 23(4):1186–95. 10.1093/ndt/gfm784 18057067

[103] Granados-Principal S Quiles JL Ramirez-Tortosa C Camacho-Corencia P Sanchez-Rovira P Vera-Ramirez L Hydroxytyrosol inhibits growth and cell proliferation and promotes high expression of sfrp4 in rat mammary tumours. Mol Nutr Food Res (2011) 55(Suppl 1):S117–26. 10.1002/mnfr.201000220 21120994

[104] Bernini R Merendino N Romani A Velotti F. Naturally occurring hydroxytyrosol: synthesis and anticancer potential. Curr Med Chem (2013) 20(5):655–70. 10.2174/092986713804999367 23244583

[105] Rafehi H Smith AJ Balcerczyk A Ziemann M Ooi J Loveridge SJ Investigation into the biological properties of the olive polyphenol, hydroxytyrosol: mechanistic insights by genome-wide mRNA-Seq analysis. Genes Nutr (2012) 7(2):343–55. 10.1007/s12263-011-0249-3 21953375 PMC3316757

[106] Emília Juan M Uwe W Hannelore D Joana MP Olives and olive oil in health and disease prevention. Cancer Chemopreventive Activity of Hydroxytyrosol: A Natural Antioxidant from Olives and Olive Oil. London: Elsevier (2010). p. 1295–300.

[107] Hsiao AF Wong MD Kanouse DE Collins RL Liu H Andersen RM HCSUS Consortium. Complementary and alternative medicine use and substitution for conventional therapy by HIV-infected patients. J Adquir Immune Defic Syndr (2003) 33(2):157–65. 10.1097/00126334-200306010-00007 12794548

[108] Wright SC Maree JE Sibanyoni M. Treatment of oral thrush in HIV/AIDS patients with lemon juice and lemon grass (*Cymbopogon citratus*) and gentian violet. Phytomedicine (2009) 16(2–3):118–24. 10.1016/j.phymed.2008.07.015 19109001

[109] Zou W Liu Y Wang J Li H Liao X. Traditional chinese herbal medicines for treating HIV infections and AIDS. Evid Based Complement Alternat Med (2012) 2012:950757. 10.1155/2012/950757 23326295 PMC3545408

[110] Lee-Huang S Zhang L Huang PL Chang YT Anti-HIV activity of olive leaf extract (OLE) and modulation of host cell gene expression by HIV-1 infection and OLE treatment. Biochem Biophys Res Commun (2003) 307(4):1029–37 10.1016/S0006-291X(03)01292-0 12878215

[111] Lee-Huang S Huang PL Zhang D Lee JW Bao J Sun Y Discovery of small-molecule HIV-1 fusion and integrase inhibitors oleuropein and hydroxytyrosol: Part I. Integrase inhibition. Biochem Biophys Res Commun (2007) 354(4):872–8 10.1016/j.bbrc.2007.01.071 17275783 PMC2790717

[112] Lee-Huang S Huang PL Zhang D Lee JW Bao J Sun Y Discovery of small-molecule HIV-1 fusion and integrase inhibitors oleuropein and hydroxytyrosol: Part II. Integrase inhibition. Biochem Biophys Res Commun (2007) 354(4):879–84. 10.1016/j.bbrc.2007.01.058 17261269 PMC1857318

[113] Crea R Method and Composition for Treatment of Inflamation and AIDS-Associated Neurological Disorders. US 0039066 A1 (2004).

[114] Gómez-Acebo E Alcamí Pertejo J Auñón-Calles D Topical Use of Hydroxytyrosol and Derivatives for the Prevention of HIV Infection. WO 067302 A1 (2011).

[115] Vissers MN Zock PL Katan MB. Bioavailability and antioxidant effects of olive oil phenols in humans: a review. Eur J Clin Nutr (2004) 58(6):955–65. 10.1038/sj.ejcn.1601917 15164117

[116] De la Torre-Robles A Rivas A Lorenzo-Tovar ML Monteagudo C Mariscal-Arcas M Olea-Serrano F. Estimation of the intake of phenol compounds from virgin olive oil of a population from southern Spain. Food Addit Contam Part A Chem Anal Control Expo Risk Assess (2014). 10.1080/19440049.2014.935961 24945796

[117] Giordano E Dávalos A Visioli F. Chronic hydroxytyrosol feeding modulates glutation-mediated oxido-reduction pathways in adipose tissue: a nutrigenomic study. Nutr, Metab Cardiovasc Dis (2014) 24(10):1144–50. 10.1016/j.numecd.2014.05.003 24984826

[118] Giordano E Dávalos A Nicod N Visioli F. Hydroxytyrosol attenuates tunicamycin-induced endoplasmic reticulum stress in human hepatocarcinoma cells. Mol Nutr Food Res (2014) 58:954–62. 10.1002/mnfr.201300465 24347345

[119] Visioli F Wolfram R Richard D Abdullah MICB Crea R. Olive phenolics increase glutathione levels in healthy volunteers. J Agric Food Chem (2009) 57:1793–6. 10.1021/jf8034429 19219997

[120] D’Angelo S Manna C Migliardi V Mazzoni O Morrica P Capasso G Pharmacokinetics and metabolism of hydroxytyrosol, a natural antioxidant from olive oil. Drug Metab Dispos (2001) 29(11):1492–8. 11602527

[121] Christian MS Sharper VA Hoberman AM Seng JE Fu L Covell D The toxicity profile of hydrolyzed aqueous olive pulp extract. Drug Chem Toxicol (2004) 27(4):309–30. 10.1081/DCT-200039714 15573469

[122] Auñon-Calles D Canut L Visioli F. Toxicological evaluation of pure hydroxytyrosol. Food Chem Toxicol (2013) 55(0):498–504. 10.1016/j.fct.2013.01.030 23380205

[123] The AIM-HIV Project. Available from: http://aim-hiv.isciii.es/

